# Mass spectrometry for the detection of potential psychiatric biomarkers

**DOI:** 10.1186/2049-9256-1-8

**Published:** 2013-06-05

**Authors:** Armand G Ngounou Wetie, Izabela Sokolowska, Kelly Wormwood, Katherine Beglinger, Tanja Maria Michel, Johannes Thome, Costel C Darie, Alisa G Woods

**Affiliations:** Biochemistry and Proteomics Group, Department of Chemistry and Biomolecular Science, Clarkson University, 8 Clarkson Avenue, Potsdam, NY 13699-5810 USA; Department of Psychiatry, University of Rostock, Rostock, Gehlsheimer Straße 20, D-18147 Germany; College of Medicine, Swansea University, Singleton Park, Swansea, SA2 8PP UK; Neuropsychology Clinic and Psychoeducation Services, SUNY Plattsburgh, 101 Broad St, Plattsburgh, 12901 NY USA

**Keywords:** Mass spectrometry, Biomarker, Psychiatry, Proteomics

## Abstract

The search for molecules that can act as potential biomarkers is increasing in the scientific community, including in the field of psychiatry. The field of proteomics is evolving and its indispensability for identifying biomarkers is clear. Among proteomic tools, mass spectrometry is the core technique for qualitative and quantitative identification of protein markers. While significant progress has been made in the understanding of biomarkers for neurodegenerative diseases such as Alzheimer’s disease, multiple sclerosis and Parkinson’s disease, psychiatric disorders have not been as extensively investigated. Recent and successful applications of mass spectrometry-based proteomics in fields such as cardiovascular disease, cancer, infectious diseases and neurodegenerative disorders suggest a similar path for psychiatric disorders. In this brief review, we describe mass spectrometry and its use in psychiatric biomarker research and highlight some of the possible challenges of undertaking this type of work. Further, specific examples of candidate biomarkers are highlighted. A short comparison of proteomic with genomic methods for biomarker discovery research is presented. In summary, mass spectrometry-based techniques may greatly facilitate ongoing efforts to understand molecular mechanisms of psychiatric disorders.

## Review

Recently, there have been increased efforts to address psychiatric disorders at the molecular level to understand the pathophysiology and molecular mechanisms involved. A major step has been the effort to discover putative biomarkers specific to psychiatric disorders [1, 2]. Biological markers (biomarkers) are measurable physiological indicators of disease or a disorder and bear a tremendous potential for diagnosis and treatment monitoring. Biomarker research employs mainly “omic” technologies (genomics, transcriptomics and proteomics) as shown for example, in the transcriptomics profiling of psychosis or mood disorders as well as the proteomic profiling of bipolar disorder [3–5]. Though genomics and transcriptomics were among the first tools used, they are being increasingly substituted by proteomics. However, all approaches have individual as well as common advantages and challenges. One collective challenge of both proteomics and genomics lies in the confirmation or validation of identified proteins [6]. Regardless of the approach, the ultimate goal is the discovery of a list of valid, reliable and selective biomarkers.

In this review, we highlight the contribution of proteomics, especially of mass spectrometry (MS) to biomarker discovery research for psychiatric disorders. Proteomics have been of considerable benefit to other disciplines, which suggests that this approach may also be of great interest to psychiatry. For example proteomic analysis is used clinically to identify bacterial subtypes in cystic fibrosis [7], cystitis [8] and septic shock [9], directing treatment. Other clinical uses of proteomics/MS include measurement of renal function [10] and newborn screening for a variety of disorders [11, 12]. Proteomic biomarker discovery holds great promise in cancer research for clinical diagnostics, based on the identification of new cancer biomarkers that might open new roads to improved diagnosis and treatment [13–15]. Phosphorylated salivary tau can be detected in people with Alzheimer’s disease using proteomic methodology, suggesting diagnostic potential [16]. These are but a few examples illustrating the clinical use and potential of MS-based proteomics.

Our intention is to speak to all involved partners in psychiatric research: psychiatrists, neuroscientists, proteomic researchers in order to bring them together to find answers to some of the questions raised here. Therefore, we first describe the basics of psychiatric disorders and of mass spectrometry, before looking at the connection between both disciplines.

### Example biomarkers in psychiatric disorders

Biomarkers are not routinely used for clinical detection of psychiatric disorders while other widespread medical conditions such as diabetes and heart disease are identified and monitored using several markers [17–19]. There have been numerous studies pointing towards genetic and epigenetic etiologies for psychiatric disorders. By nature, psychiatric disorders may have a concerted pathway that orchestrates the down-regulation of multiple genes through epigenetic mechanisms. For instance, DNA methylations or histone modifications in combination with GABAergic and glutamatergic gene promoters are proposed to be critical elements in the pathogenesis of schizophrenia and bipolar disorder as demonstrated by decreased protein levels of GABA-ergic neuronal markers (reelin, RELN and glutamic acid decarboxylase 67, GAD67) [20–23]. Additionally, it has been suggested that polymorphisms in *ADRA2A* (alpha-2A adrenergic receptor), *DRD3* (dopamine receptor D3), *DBH* (dopamine β-hydroxylase) and *SNAP-25* (25 kDa synaptosomal-associated protein) are individually or collectively risk factors for schizophrenia [24–27]. As just discussed, there are many potential genetic sources from which biological markers could be retrieved in psychiatric disorders. However, non-genomic biomarkers, such as protein/peptide biomarkers, also hold promise.

Due to their broad variety, psychiatric disorders have been hypothesized to be associated with the dysfunction of many biological pathways and networks. For example, impairment of the corticotropin releasing factor (CRF) system is proposed to contribute to symptoms in psychiatric disorders such as depression, obsessive compulsive disorder, post-traumatic stress disorder and substance use disorders [28–32]. Depression has been associated with low plasma levels of antioxidants such as vitamin E, zinc, glutathione (GSH), coenzyme Q10, selenium [33–38].

Dopamine (DA) and noradrenaline (NE) have been proposed to play a major role in modulating high-level executive functions that are impaired in attention-deficit/hyperactivity disorder (ADHD), for example planning and attention, functions related to fronto-striato-cerebellar circuits (ADHD) [39]. Dopaminergic disturbances have also been posited to underlie the hyperactivity observed in ADHD, likely along with other neurotransmitter systems [40]. Reflecting a possible disturbance in the dopaminergic system, dopamine transporter (DAT) levels are increased in individuals with ADHD [39, 41]. A recent meta-analysis confirms that peripheral markers related to the dopaminergic and noradrenergic systems (as well as additional markers such as zinc and cortisol), may be of future use for ADHD diagnosis and treatment [42].

A current common approach in mass spectrometric characterization of proteins is differential gel-based quantitative proteomics [43, 44]. It consists of separating samples, (from subjects with disorders and unaffected controls), by gel electrophoresis (usually 2D-gel electrophoresis). Gel bands are then analysed by tryptic digestion and LC-MS/MS for protein identification and quantification. Results are often validated by western blot or ELISA. Using this approach (2D-PAGE and LCQ DECA XP PLUS ion trap mass spectrometer), Ditzen and colleagues identified glyoxalase I (GLX1) and enolase phosphatase (EP) as protein markers that could be risk markers for anxiety in a mouse model of trait anxiety [45]. A separate study utilized this technique to identify 59 potential biomarkers in cerebral cortex and 11 in amygdala in post-mortem brain tissue from suicide victims [46]. Several of these proteins were already proposed as psychiatric protein biomarkers. In another study, focusing on major depressive disorder (MDD) in a rat model, 27 potential protein markers with roles in neurogenesis, oxidative metabolism, transcription and signal transduction, were identified by 2D-gel and MALDI-TOF-MS [47]. Shotgun proteomics (explained later) was also employed for the study of brain tissue of samples from MDD patients using SDS-PAGE and nanoHPLC-MS^E^ (Q-TOF MS) and produced a possible means for categorizing different subtypes of MDD patients based on proteomic profile patterns [48]. These protein fingerprints resulted from significantly, differentially-expressed proteins between subgroups of MDD patients (with and without psychosis) as well as between MDD subjects and healthy controls. Differentially-expressed proteins between MDD patients and healthy controls were those involved in metabolism, transport, cell communication and signaling, cell growth and maintenance, protein metabolism and regulation of nucleic acid metabolism. The use of biomarkers to diagnose depression is of great interest and promise, particularly to direct the selection of therapies. However, further studies are needed before proteomics can be used clinically for depression [49].

The discovery of potential biomarkers for depression could be expanded to other related psychiatric disorders since depression is comorbid with many psychiatric, neurodegenerative and general medical conditions [28]. A proteomic study reported the association of protein markers of MDD with neurodegenerative diseases such as Huntington and Alzheimer as well as schizophrenia confirming some sense of commonality among neurological and psychiatric disorders at the molecular level [27]. Interestingly, this connection is already observed at the symptomatic level as neuropsychiatric disorders overlap in their symptoms. In this regard, there is a need for identification of a signature of a set of biomarkers rather than relying on a few markers [6, 50].

### Mass spectrometry

Proteomics is a fast-rising and promising field with new developments and improvements still taking place. As mentioned, one core method in proteomics is MS. For MS analysis of proteins, proteins are initially separated (biochemically fractionated) via a variety of methods, for example, electrophoresis or chromatography [2]. Once fractionation has been performed, proteins are analysed by MS. A mass spectrometer is made of three main parts: ionization source, mass analyser and detector. The protein sample is ionized using an ionization source. The sample then travels through a mass analyser according to the mass over charge (m/z). The ionized sample then hits the detector, where spectra are recorded. Spectra serve as protein “fingerprints” that can be used to identify proteins, for example. For a more detailed description of MS tailored to those working in psychiatric research, the reader may refer to Woods et al., 2012 [2].

Due to high-performing MS instruments, simplified analytical workflows and versatile data analysis, mass spectrometry is applicable to almost every area of the life sciences and potentially even far beyond [51–54]. The two most common ionization methods are electrospray ionization (ESI) and matrix-assisted laser desorption/ionization (MALDI). Currently a multitude of analyzers exist for different types of applications, including, quadrupole (Q), time-of-flight (TOF) and ion trap (IT). Traditionally, MALDI sources are coupled with TOF or TOF/TOF mass analyzers due to their pulse mode of action.

In shotgun proteomics, liquid chromatography (LC) is coupled to mass spectrometry for identification of proteins. In this case, ESI is the ionization mode preferred for the characterization of biomolecules, ionic and very labile organic and organometallic compounds though LC-MALDI-MS is also an eventuality [55, 56]. Bottom-up proteomics represents the case where samples are first digested to generate peptides which can then be analyzed by mass spectrometry while top-down proteomics designate a method where the mass of the entire protein is being measured by a mass spectrometer followed by its sequencing. The main advantage of LC-MALDI-MS over LC-ESI-MS is the robustness of LC-MALDI-MS in resisting to very harsh LC conditions (lower suppression effects) and the high mass-to-charge (m/z) range of the TOF mass analyzer. The disadvantage of LC-MALDI-MS is the difficulty of spotting directly from the LC apparatus. However, LC-MALDI is only rarely used.

With MS, proteins can be identified by measuring the m/z of gas phase ions. We distinguish between gel-based, one-dimensional and two-dimensional gel electrophoresis mass spectrometry (1-DE, 2-DE and MS) [57–64] and non-gel based liquid chromatography/mass spectrometry (LC/MS). In 2-DE, proteins are separated in a first dimension according to their charge or isoelectric point (pI) and in a second dimension depending on their molecular weight [65, 66]. For visualization of protein bands, the gel can be stained either with the dye coomassie blue or silver stained or by fluorescently labeling the samples before 2-DE. For mass spectrometry analysis, gel bands are removed, and undergo several other steps to generate peptides in bottom-up and shotgun proteomics [51, 53, 67, 68]. Compared to LC/MS, 2-DE methods are more reproducible and robust. However, 2-DE methods are very labor-intensive and not very suitable for hydrophobic, very large or small, basic proteins and complex sample mixtures. However, the weaknesses of 2-DE methods represent the strengths of LC/MS-based methods. LC-MS methods are appreciated for their ability in the analysis of very complex protein samples and extreme proteins (e.g. membrane proteins) and therefore offer better proteome coverage in comparison to 2-DE-based techniques.

For quantification, a label-free or label-based [69] approach can be selected. In general, quantitation is carried out with internal standards which are added to the sample prior to any sample preparation step in order to exclude any variations resulting from the sample preparation. Non-gel, label-based approaches consist in labeling peptides prior to LC separation using mostly the three following techniques: 1) isobaric tags for relative and absolute quantitation, iTRAQ (iTRAQ^®^ Applied Biosystems, AB Sciex, Foster City, CA, USA) [70, 71]; 2) isotope-coded affinity tags, ICAT [72] and 3) stable isotope labeling of amino acids in cell culture, SILAC [53, 73, 74]. These internal isotopically labeled analogs have the same physico-chemical properties, meaning same retention time, fragmentation pattern and extraction efficiency as their endogenous sample-intrinsic counterparts but differ in their mass shift as a result of the incorporation of heavy or light isotopes in their structure. However, isotopically labeled analogs present some limitations due to their targeted chemistry (e.g. cysteines for ICAT, intensive sample separation and longer MS run time). In label-free approaches (e.g. spectral counting), quantitation is based on the number of spectra generated for a reference protein or peptide. Though mostly reported for neurodegenerative proteomic studies [71, 75, 76], these quantitative techniques can also be extended to psychiatric disorders. Another MS-based quantitation approach that is commonly applied is the multiple-reaction monitoring (MRM) technique that is possible on triple quadrupole, linear ion trap MS instruments. MRM is a robust multiplexed assay for the accurate and sensitive determination of protein expression levels and post-translational protein modifications [77]. Instrument-wise, MALDI is typically coupled to QqQ mass analyzers resulting in fast and sensitive quantitation [78] that is thought to be able to challenge established LC-ESI-MS methods in term of linearity, limit of quantitation, precision and accuracy [79].

Due to the considerable amount of data generated during a proteomics experiment and the need to extract as much information as possible from these data, the field of bioinformatics has become an important tool in the discovery of biomarkers by proteomics methods and has been further improved recently [80, 81]. The same can be said of online protein databases (Expasy or HPRD), search engines (X! Tandem, MASCOT, Sequest), data management repositories (PRIDE, GPMdb), data exploration and mining tools (Ingenuity pathways analysis IPA, GOMiner, ProteinLounge, Scaffold, ProteinLynx Global Server). To date, proteomics has been more frequently employed in neurodegenerative disorders than psychiatric [82] and can and should be expanded to psychiatric disorders [4].

### Comparison of MS-methods with classical biomarker discovery techniques

With the completion of the Human Genome Project and the extensive progress made in the field of genomics together with the possible genetic etiology of some psychiatric disorders, genetics and molecular biology represent one of the predominant methods of psychiatric biomarker discovery. Using chromosome microarray with probes for both copy number variants and single nucleotide polymorphisms (SNPs), determination of disorder-underlying genes and segmental deletions or duplications is possible. Newly developed methods such as exome sequencing enable detection of SNPs, gene regulatory sequences and mutations of protein coding genes that could be the root of a psychiatric disorder. However, genomics have some limitations. For example, genomic methods cannot distinguish splice variants or proteins with post-translational modifications.

Though genomics was originally the state of the art discipline for psychiatric biomarker discovery; one can notice the shift that has been taking place in the last decade from genomics- to proteomics-based techniques [26, 83–91]. This shift may be due in part to the fact that changes at the mRNA level are not necessarily reflected at the protein level [92, 93]. Changes at the protein level may reflect disorder processes that cannot be detected at the genomic level. Genomic and proteomic information may be complementary, contributing to more of a systems approach to understanding the biology of psychiatric disorders [1].

One advantage of genetics in biomarker discovery is their superior high-throughput. The amount of mRNA or SNPs that can be analyzed at once is in the tens of thousands for genetic studies compared to few thousand for proteomics. Also, due to the non-translation of some transcripts and the non-secretion of some proteins, transcript rather than protein levels are chosen to be monitored. Finally, the isolation of RNA from blood does not require refrigeration in contrast with plasma and serum samples and is RNA less susceptible to degradation [94].

In some cases, it would be interesting to pursue both genomics and proteomics works to seek confirmation or complementarity of results. Genomic markers and protein markers may ultimately play interesting and possibly complementary roles in psychiatric diagnosis, since genomic information may indicate disorder susceptibility. Due to the ever-changing nature of proteins, proteomic information is more likely to allow for monitoring of certain aspects of a disorder, such as severity and response to treatment. Multiple markers obtained using different data-collection approaches (such as genomic, proteomic, neuroanatomical, brain activity patterns, etc.) comprising a biomarker signature, could aid differential diagnosis, particularly since individual markers are likely to overlap amongst psychiatric syndromes. Indeed, a systems approach to neuropsychiatric disorders has been recently proposed, which would address the multifactorial aspects and complexity of psychiatric problems [1], which would include a consideration of environment, experience and behavior as well as the data measurements listed above. Mass spectrometry and proteomics have the potential to be major components of such an approach.

## Discussion

There are many challenges and stumbling blocks that need to be addressed for mass spectrometry to develop its full potential in psychiatric biomarker research. It is now well known that more than 20,000 genes are expressed in the brain and more than 300 potential post-translational modifications have been determined [95]. Further, there is a wide dynamic range in regard to the relative abundance of proteins in brain cells and tissues or in response to external factors.

Consequently, the exact molecular etiology as well as the distinct description of most psychiatric disorders is still not well defined. Therefore, there is a dire need for discovery of new proteins [54, 96–99] and of molecular biomarkers to categorize, prognosticate, monitor or treat psychiatric disorders [44]. Another challenge is the existence of adequate model systems that mimic exactly the pathophysiological situations taking place in the brain. It is difficult to assess cognitive and neuropsychiatric symptoms such as hallucinations or suicidal ideations in any model organisms. As for human studies, the problem resides in the availability of well-characterized human material taken according to international standard protocols. Further, large pools of samples are required for high-throughput analyses and data, and also due to individual variability. Regarding databases, software or programs used in genomics and proteomics there is a risk that genes and proteins that are well-characterized and described will have a higher detection rate than those with unknown function.

Mass spectrometry can and will most likely play a major role in the identification of psychiatric biomarkers. Several recent developments and innovations have occurred in the field of mass spectrometry in particular and proteomics in general. These include increased machine sensitivity allowing detection of proteins found at low concentrations and improved software allowing not only protein detection, but even the analysis of an entire protein pathway. These advancements make this discipline of increasing potential utility to further the understanding of molecular mechanisms underlying the pathophysiology of psychiatric disorders. Several possible advantages of mass spectrometry exist with regard to possible diagnostic use. For one, mass spectrometry can identify all proteins in a sample, whereas other techniques require that the protein of interest is targeted. Second, analysis of biomaterials such as blood, saliva or urine is convenient and non-invasive (in the case of saliva and urine). Third, the sensitivity of recently developed machinery is extremely high, increasing the potential that peripheral bodily fluids may actually reflect central nervous system protein contents. Finally, protein marker changes could potentially precede behavioral changes, giving an earlier indication of whether a treatment is working. This may be particularly useful in psychiatry, since treatment effects are often not immediately measurable in people with psychiatric problems. The determination of proteome fingerprints or profiles specific to a disorder could open the door to a new way of discovering biomarkers for the diagnosis, prognosis, monitoring and treatment of psychiatric disorders.

Scientists have in some instances thought of biomarkers as a single biomolecule which is differentially expressed in a unique disease. However, as already mentioned above, some psychiatric disorders share similar symptoms and molecular pathways. It may therefore be more appropriate to concentrate on a biomarker signature of multiple molecules which can be altered in order to monitor and identify a psychiatric disorder [100]. Besides examining peripheral and bodily fluids such as the cerebrospinal fluid (CSF) or serum, one could consider, (if available), the use of the primary brain tissue for proteomics experiment. However, to study brain tissue requires a biopsy (available mostly for cancerous tissues or epilepsy) or post-mortem tissue (quality can be sometimes questionable). Further, psychiatry-based proteomics could also investigate protein-protein interactions and protein post-translational modifications (PTMs) as well as the whole proteome. As for the design of the studies, care should be taken to consider high variability in age, gender, demographics, race, postmortem interval, drug treatment, comorbidities and other influencing factors [101]. Another critical point is the evaluation, interpretation and follow-up of results of proteomics studies. Traditionally, validation has been performed with biochemical methods such as western blotting and ELISA. However, these methods are limited by their low high-throughput capability (mostly western blotting) as well as the high cost and difficulty associated with the production of very specific antibodies (ELISA). Fortunately, mass spectrometric methods such as MRM are strong alternatives for the validation of proteins identified in high-throughput proteomics experiments.

## Conclusions

The application of mass spectrometry-based methods opens a new avenue for the investigation of psychiatric disorders with the clear objective of understanding and identifying altered protein pathways as well as uncovering psychiatric biomarkers for diagnosis, prognosis and treatment monitoring. Eventually mass-spectrometry may facilitate treatment of psychiatric disorders through the identification of therapeutic targets.
